# Supplementary material for: Spatially resolved multiomics on the neuronal effects induced by spaceflight in mice
Source: Nat Commun. 2024 Jun 11;15:4778. doi: 10.1038/s41467-024-48916-8 (PMC11166911; doi:10.1038/s41467-024-48916-8)
Supplement: Supplementary file 1 — Supplementary Information [file 41467_2024_48916_MOESM1_ESM.pdf]

Supplementary Information

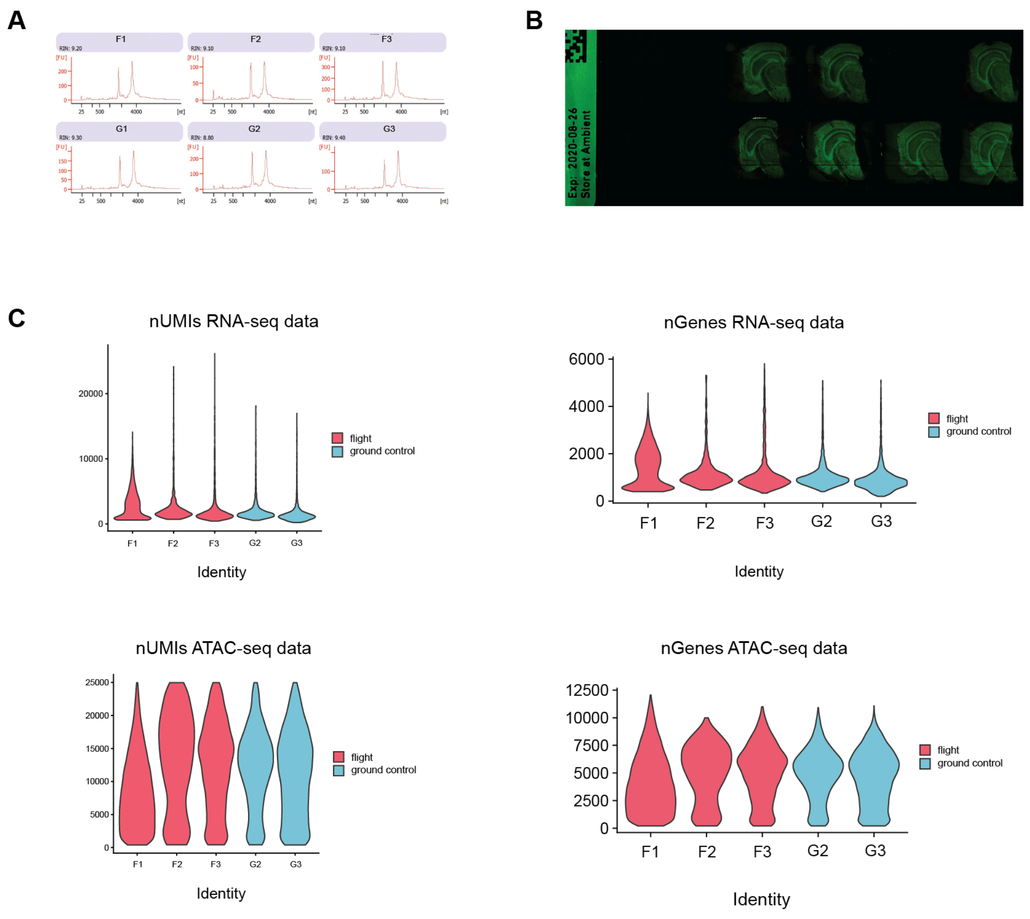

Supplementary Figure 1: A, Bioanalyzer traces showing RIN values for all brain samples. **B**, Fluorescent footprints of brain tissue section obtained from Visium Tissue Optimization protocol by 10x Genomics (for detailed protocol see: UserGuide_RevB, CG000238), each section footprint represents different times tested during permeabilization step, empty position represents negative control, section untreated with permeabilization mix. **C**, Distributions of unique molecules and genes detected per nucleus per sample in single nuclei RNA-seq multiomics dataset (RNA-seq on top, and ATAC-seq on bottom) visualized as violin plots colored by sample condition (i.e., flight, and ground control).

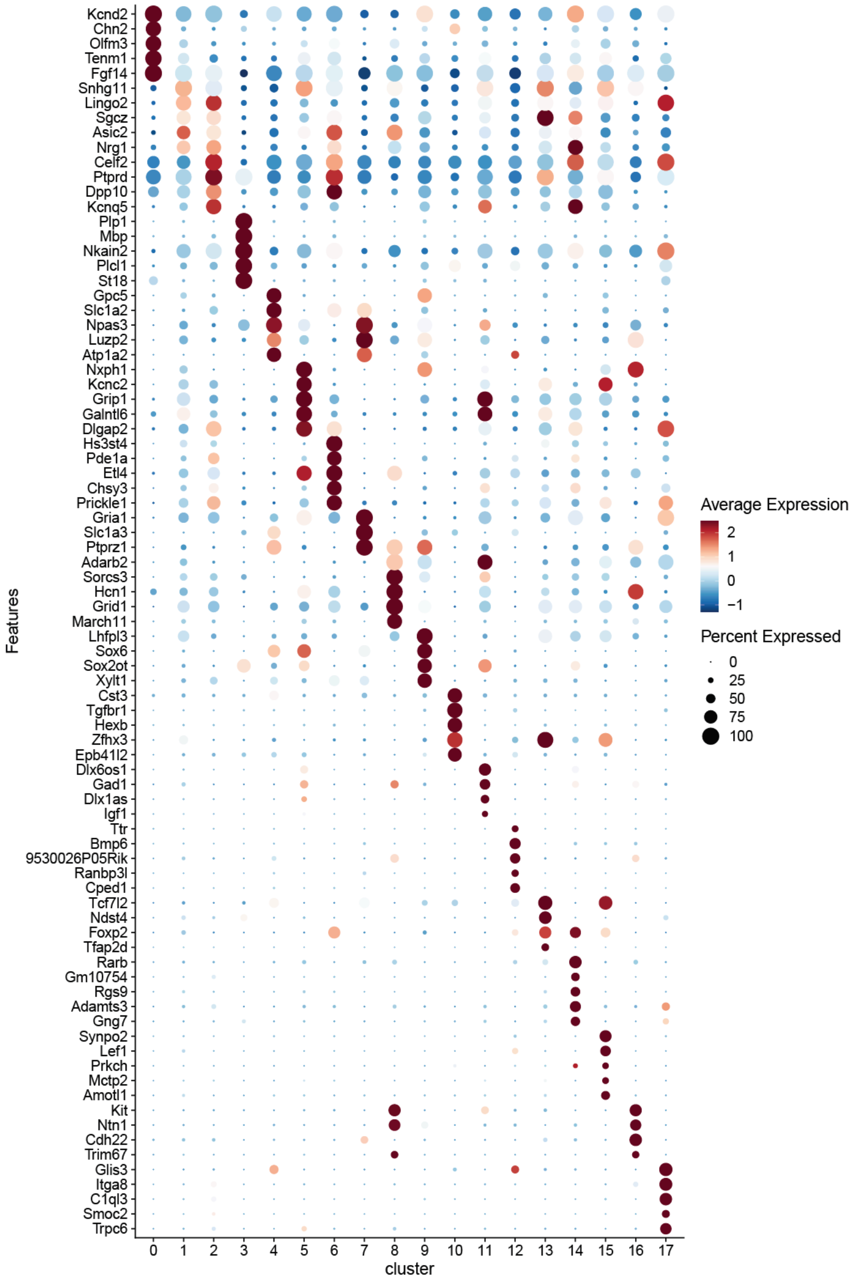

Supplementary Figure 2: Marker genes for each multiomics cluster visualized as Dot Plot. These marker genes were used for annotation of the multiomics clusters based on key functions as well as cell types. Two-sided Wilcoxon's rank sum test was done to estimate for adjusted *p*-values (adjusted p-value < 0.05).

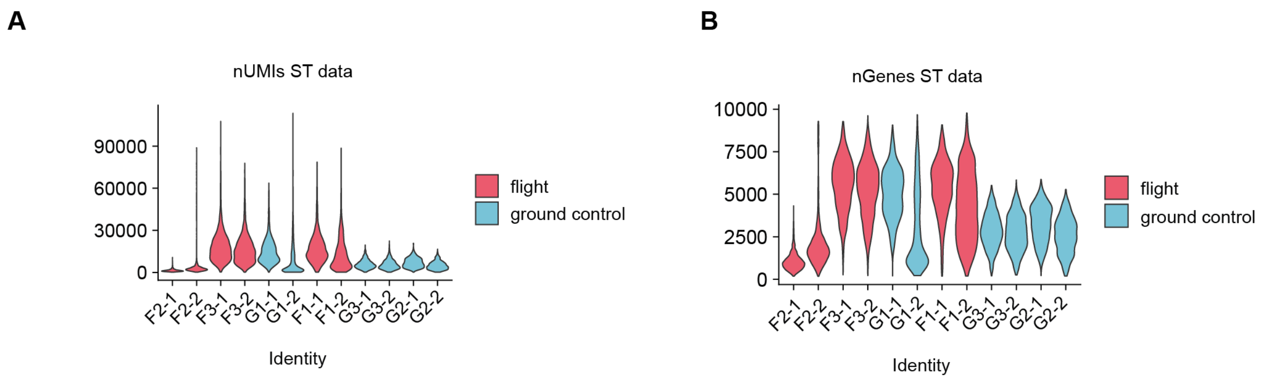

Supplementary Figure 3: A, Distributions of unique molecules (nUMIs) per spot per tissue section in ST dataset visualized as violin plots colored by sample condition (i.e., flight, and ground control). **B,** Distributions of genes (nGenes) per spot per tissue section in ST dataset visualized as violin plots colored by sample condition (i.e., flight, and ground control).

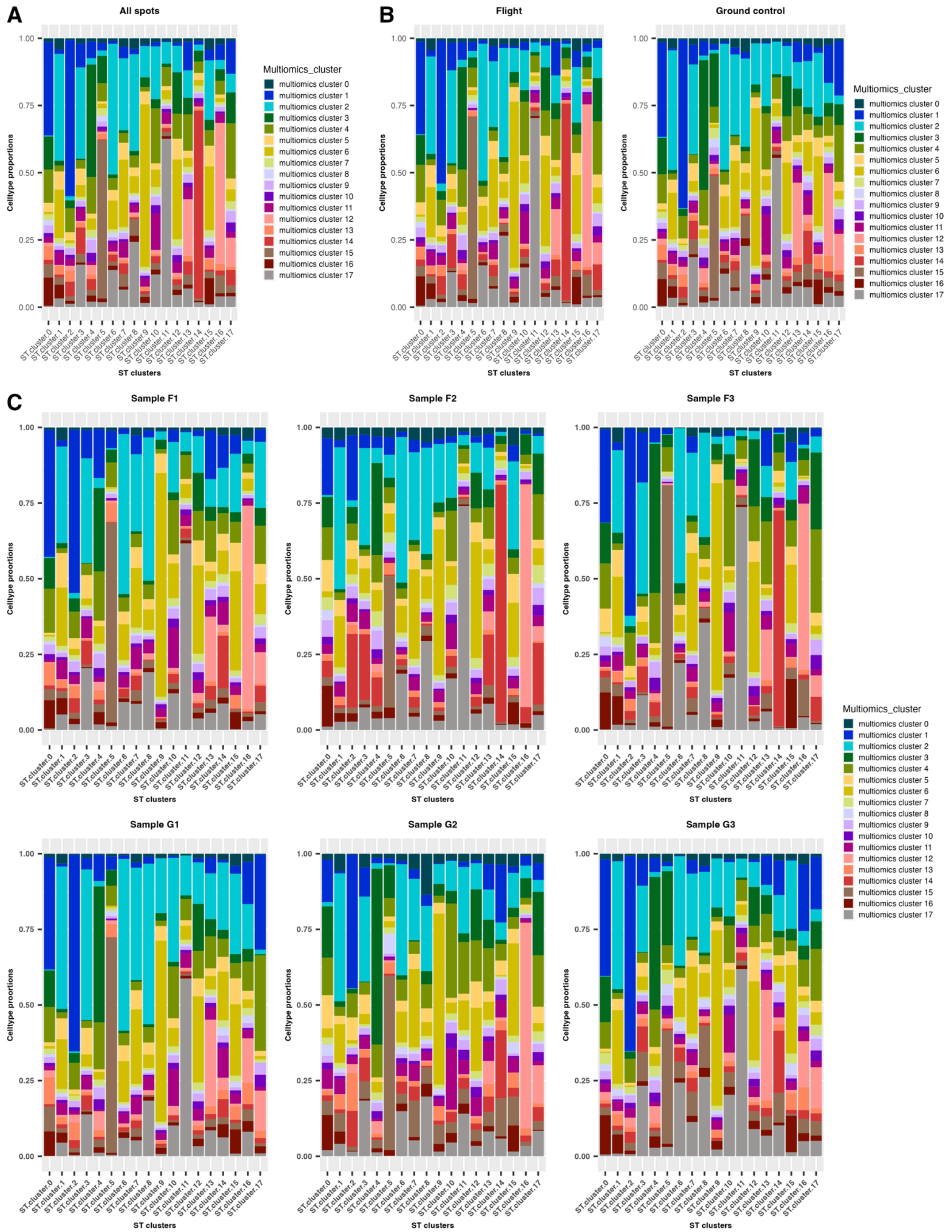

Supplementary Figure 4: A, Barplot showing all the proportions of multiomics clusters (multiomics cluster 0-17) in each ST cluster found from Stereoscope deconvolution. **B,** Barplot showing multiomics cluster proportions in each ST cluster separated by sample condition (Flight

samples on the left and Ground control on the right). **C,** Barplot showing multiomics cluster proportions in the ST clusters displayed separately for each individual sample. F1, F2, F3 - Flight; G1, G2, G3 - Ground control.

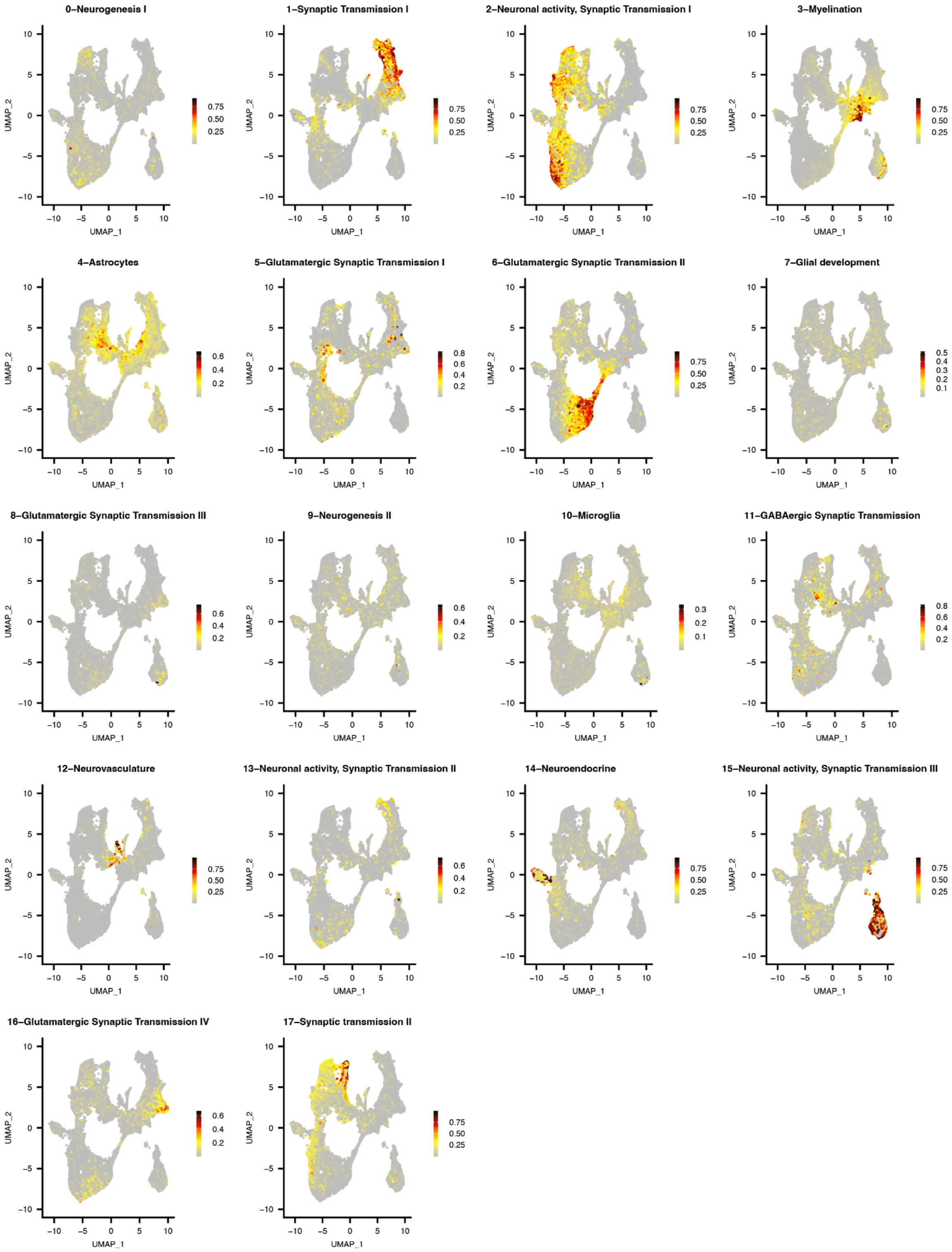

Supplementary Figure 5: Distribution of the multiomics clusters identified from Stereoscope deconvolution in the matching ST dataset, visualized as UMAP plot. The legend corresponds to

the proportion values of each multiomics cluster calculated by Stereoscope for each ST spot, ranging from 0 (no expression) to 1 (complete expression).

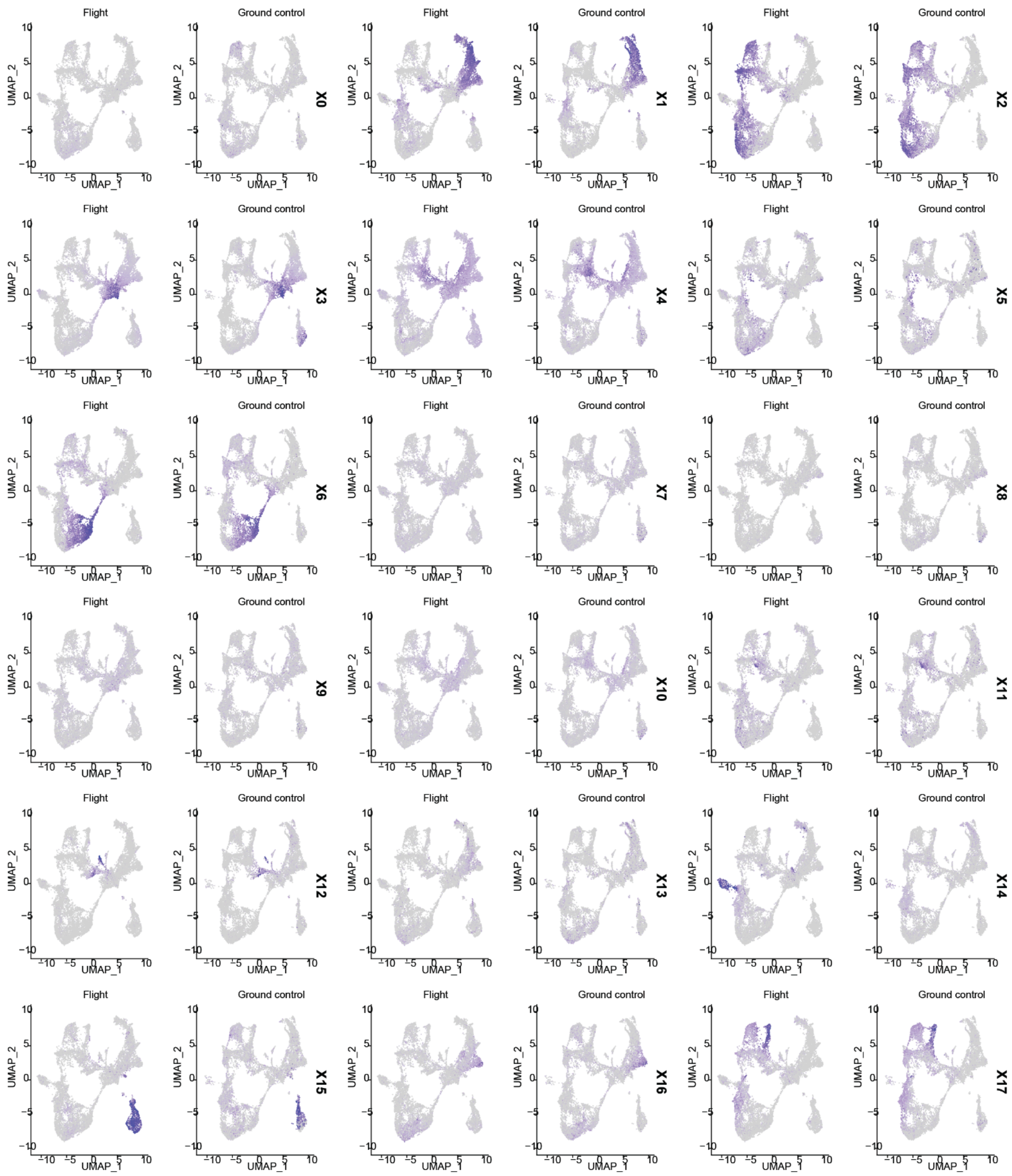

X0: 0-Neurogenesis I
X1: 1-Synaptic Transmission I
X2: 2-Neuronal activity, Synaptic Transmission I
X3: 3-Myelination
X4: 4-Astrocytes
X5: 5-Glutamatergic Synaptic Transmission I
X6: 6-Glutamatergic Synaptic Transmission II
X7: 7-Glial development
X8: 8-Glutamatergic Synaptic Transmission II
X9: 9-Neurogenesis II
X10: 10-Microglia
X11: 11-GABAergic Synaptic Transmission
X12: 12-Neurovasculature
X13: 13-Neuronal activity, Synaptic Tranmission II
X14: 14-Neuroendocrine
X15: 15-Neuronal activity, Synaptic Transmission III
X16: Glutamatergic Synaptic Transmission IV
X17: 17-Synaptic Transmission II

Supplementary Figure 6: Distribution of the multiomics clusters (X0-X17) identified from Stereoscope deconvolution in the matching ST dataset, visualized as a UMAP plot in split-view

format separating the flight and ground control samples. Cluster annotations for the multiomics clusters (X0-X17) are also stated at the bottom of the figure.

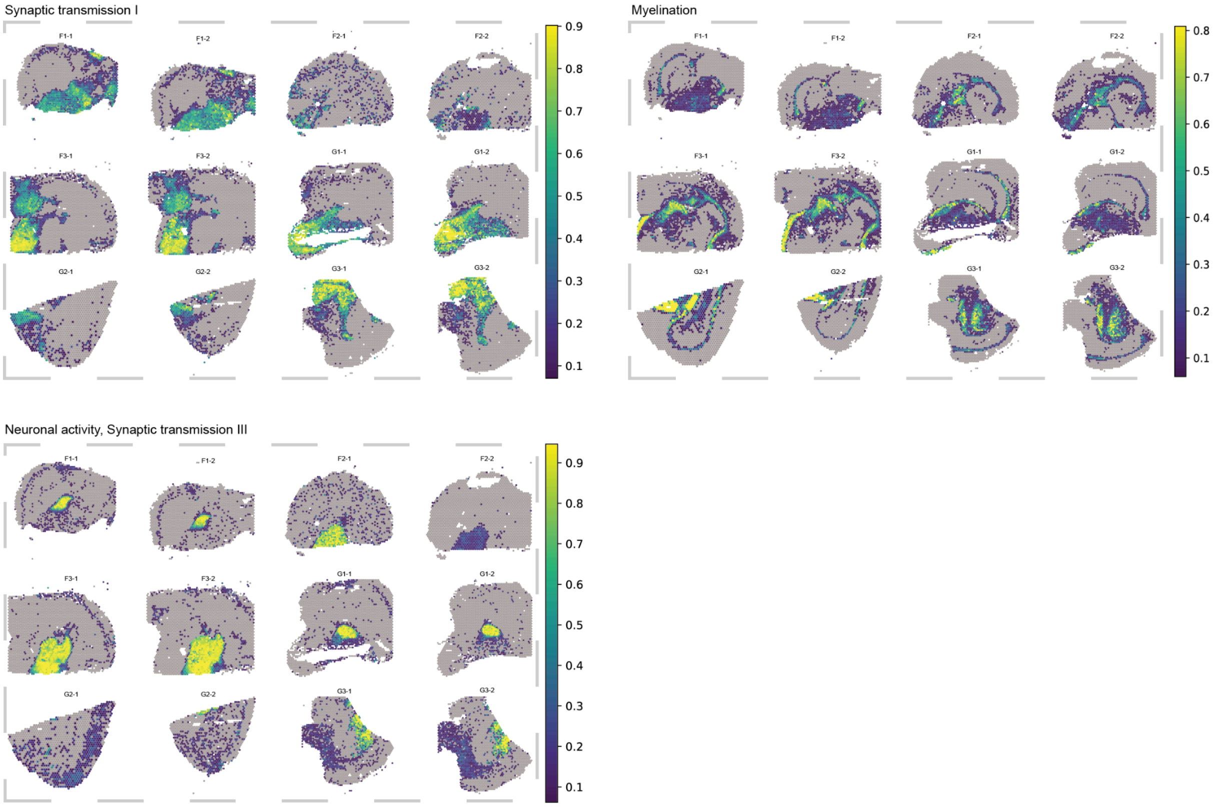

Supplementary Figure 7: Spatial localization of multiomics clusters 1, 3 and 15 (Synaptic Transmission I, Myelination and Neuronal activity, Synaptic Transmission III) visualized on all ST brain sections.

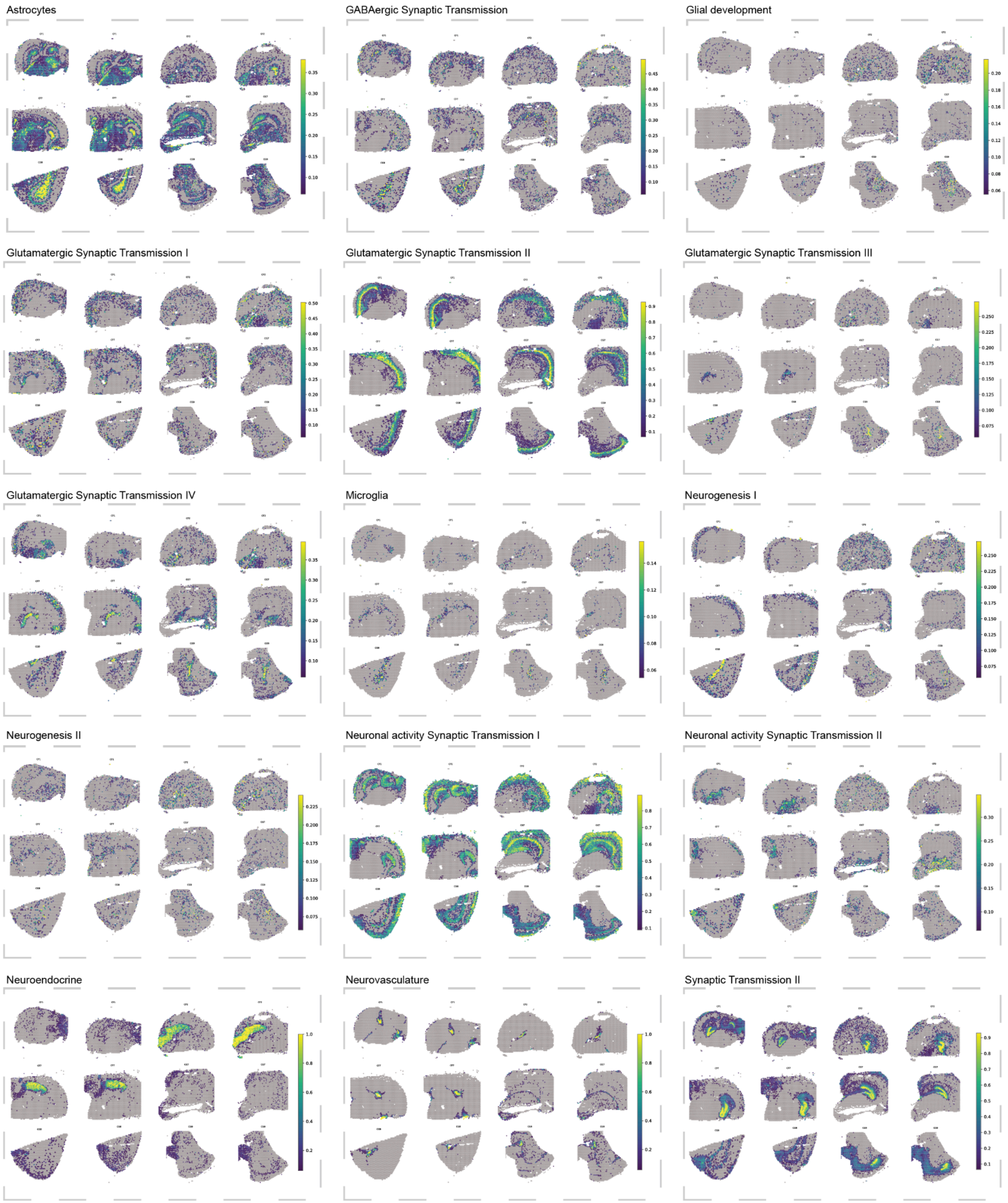

Supplementary Figure 8: Spatial localization of the remaining multiomics clusters visualized on all ST brain sections.

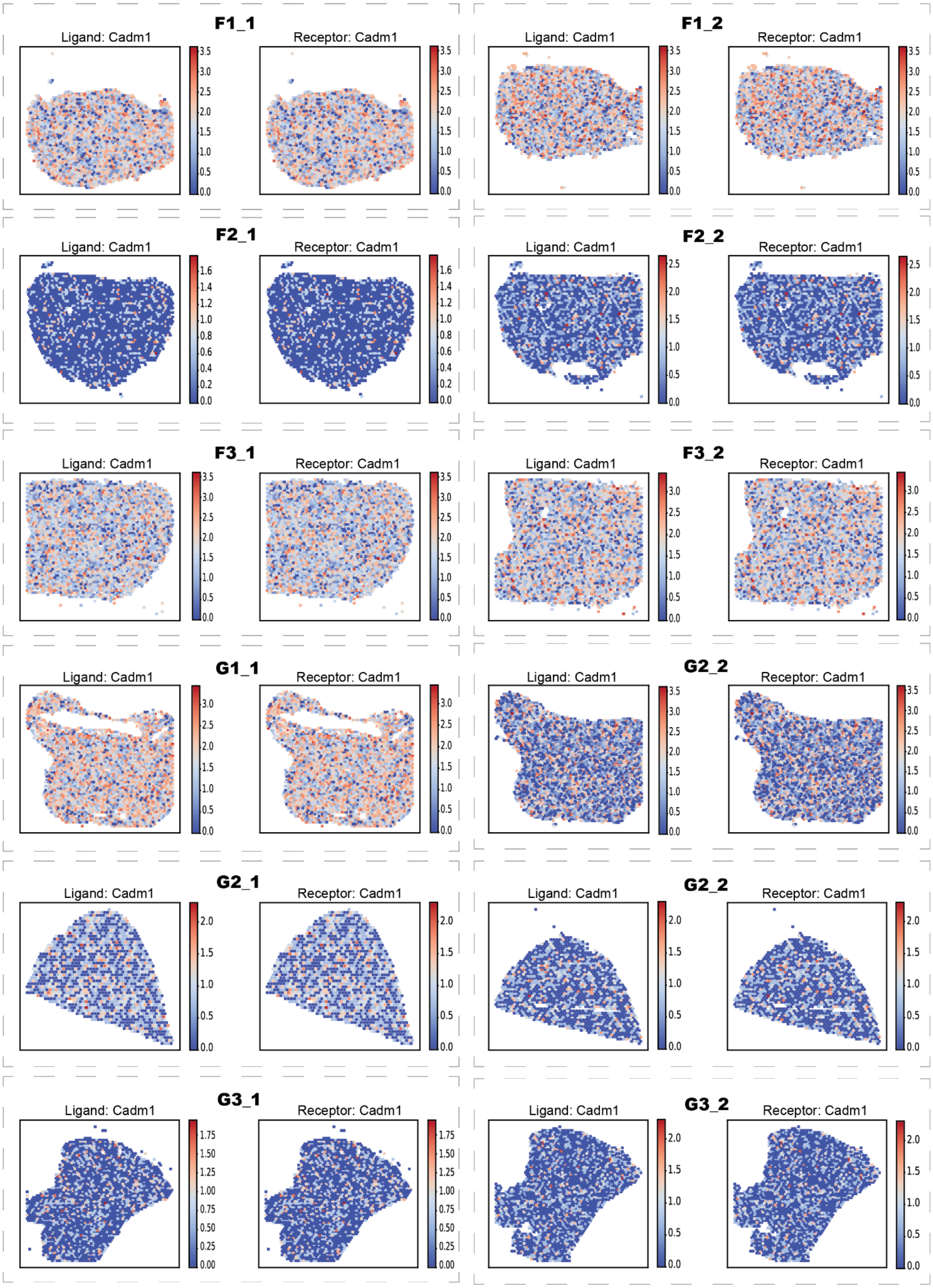

Supplementary Figure 9: Local spots selected by SpatialDM for ligand-receptor pair CADM1-CADM1 across all 12 ST brain sections, shown as an example heatmap.

Glycolysis_gluconeogenesis - ST

Heatmap of log2 FC flight vs ground

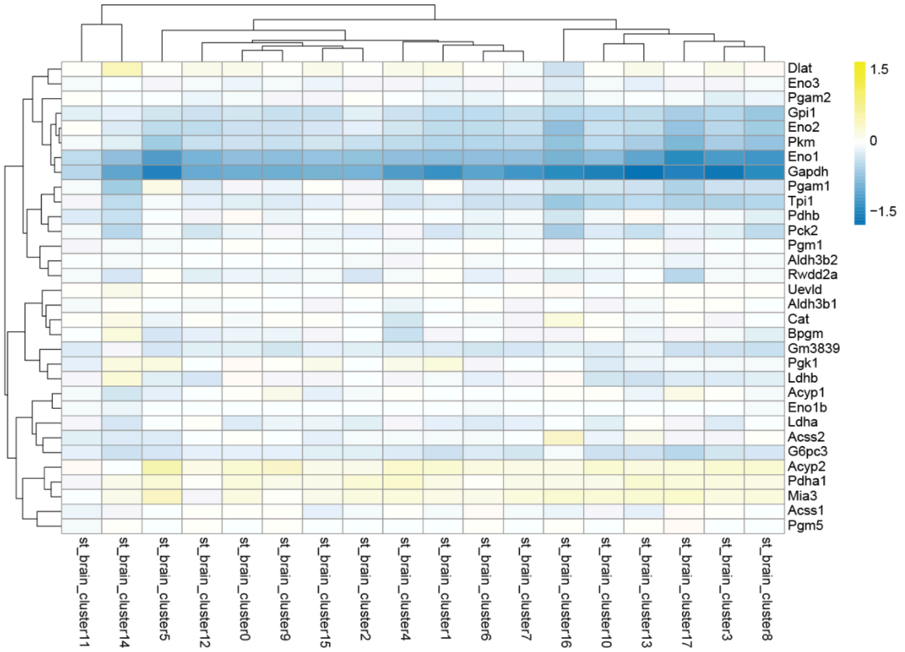

Glycolysis_gluconeogenesis - multiomics

Heatmap of log2 FC flight vs ground

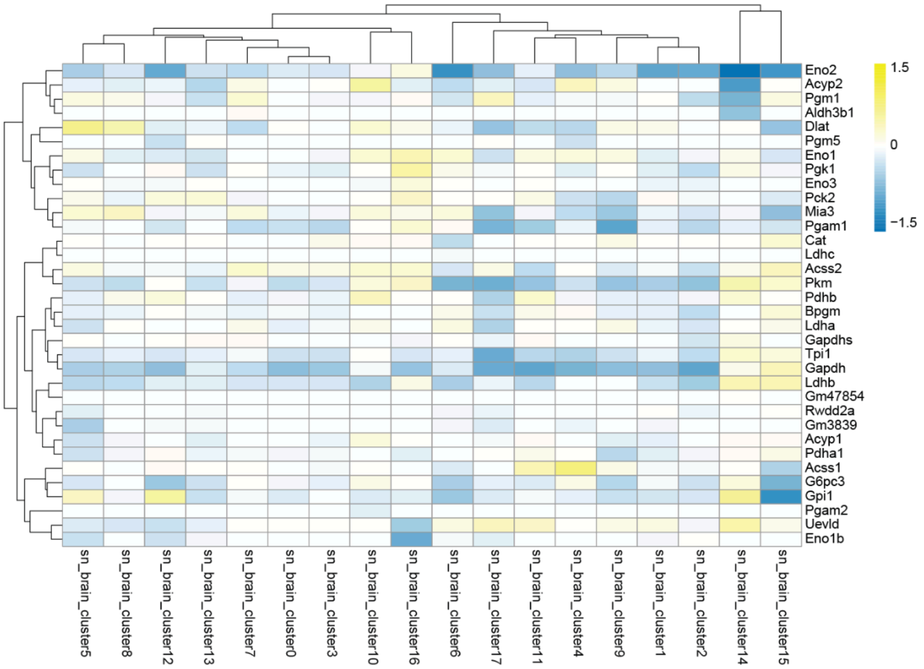

Supplementary Figure 10: Heatmap showing relative fold change differences (log2FC values) between flight and ground control samples in glycolysis and gluconeogenesis pathways in both ST and multiomics datasets. st_brain_cluster: ST Cluster, sn_brain_cluster: multiomics cluster.

Fructose and mannose metabolism - ST
Heatmap of log2 FC flight vs ground

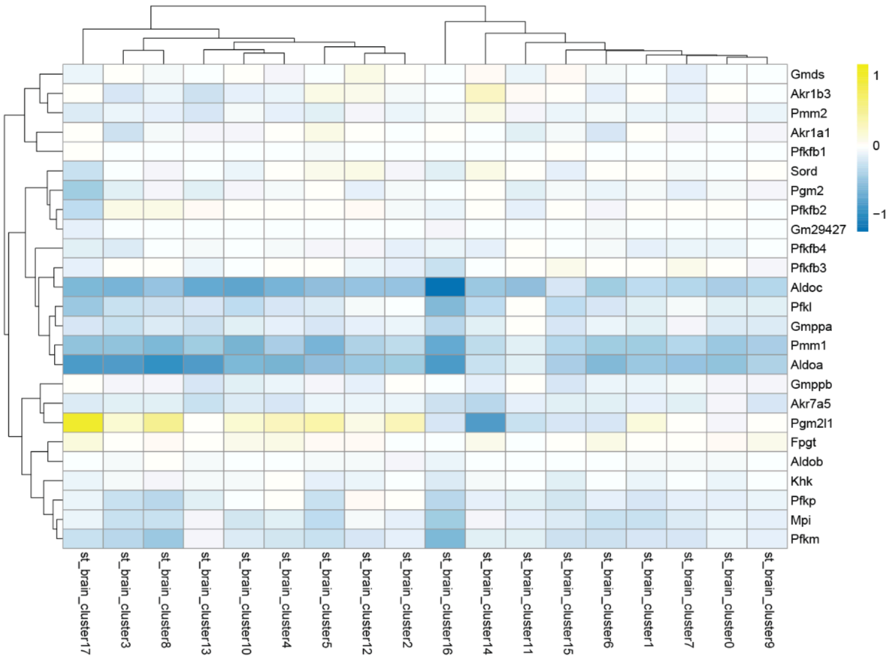

Fructose and mannose metabolism - multiomics
Heatmap of log2 FC flight vs ground

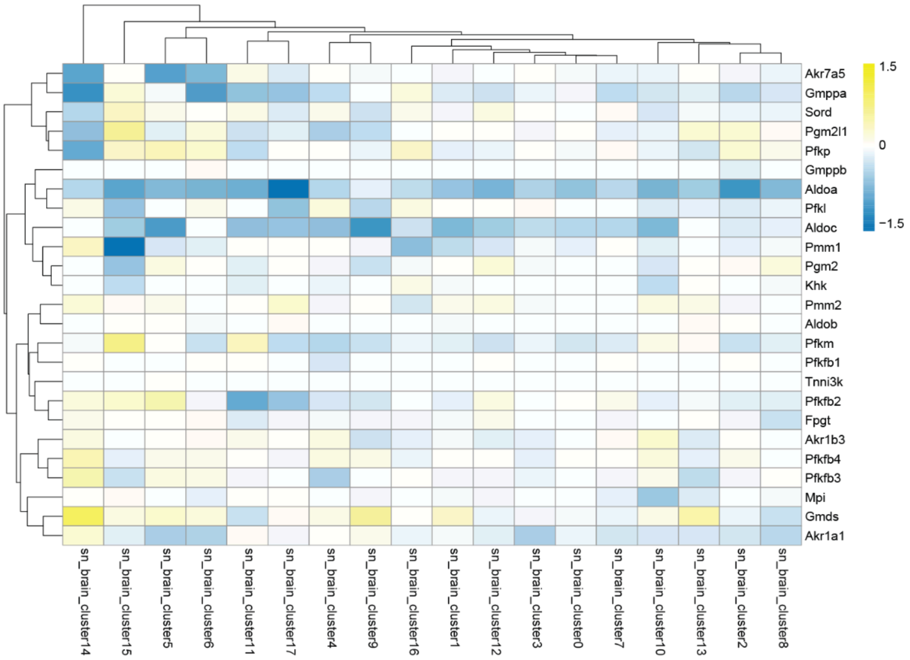

Supplementary Figure 11: Heatmap showing relative fold change differences (log2FC values) between flight and ground control samples in fructose and mannose metabolism pathway in both ST and multiomics datasets. st_brain_cluster: ST Cluster, sn_brain_cluster: multiomics cluster.

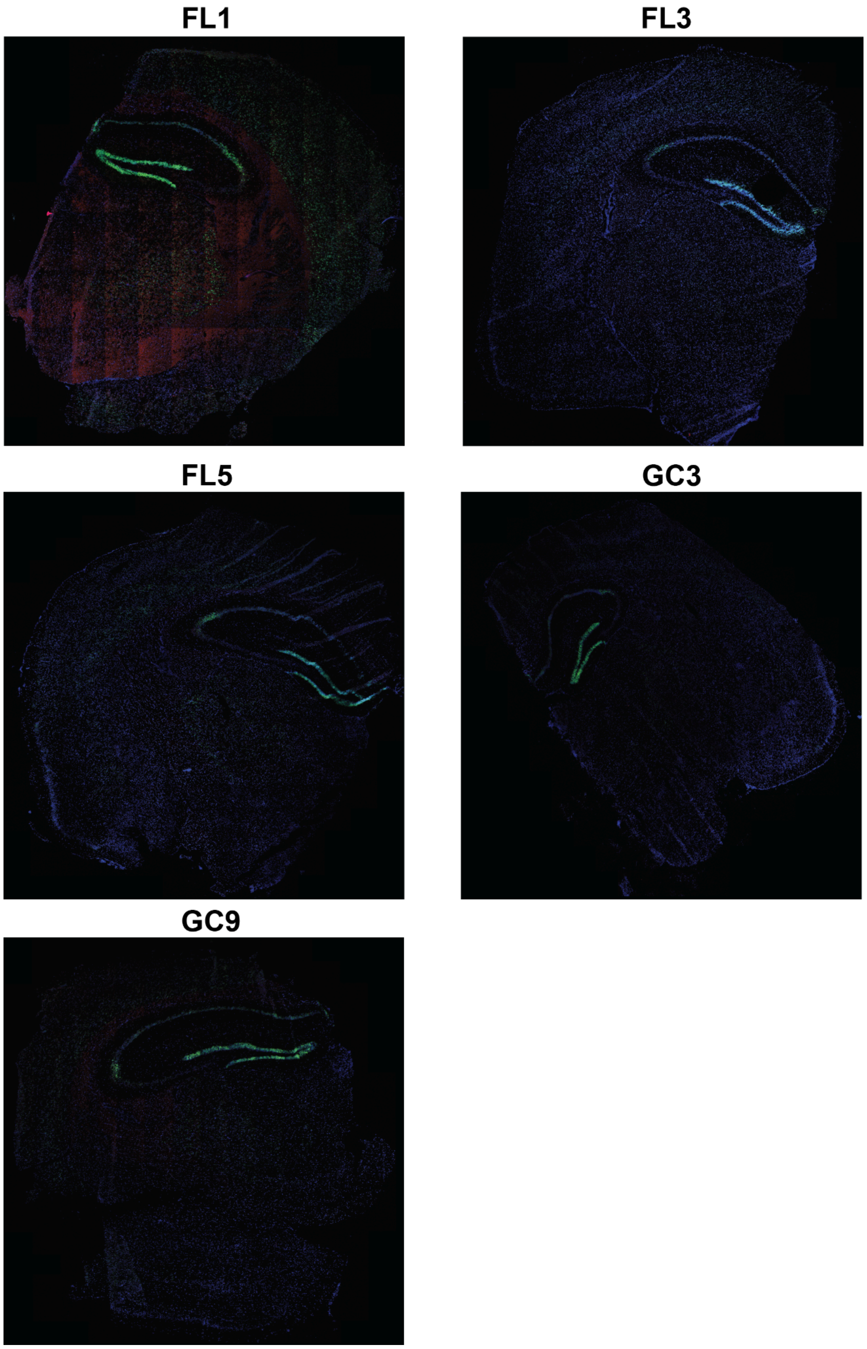

Supplementary Figure 12: RNAscope fluorescence images of the whole brain sections for the five brain samples used in the validation experiments. FL1, FL3, FL5 refer to the spaceflight samples and GC3, GC9 refer to the ground control samples. Nuclei are visualized via three fluorescence channels: blue for DAPI, green (code 488) for gene of interest Adcy1, and red (code 555) for gene of interest Gcp5.

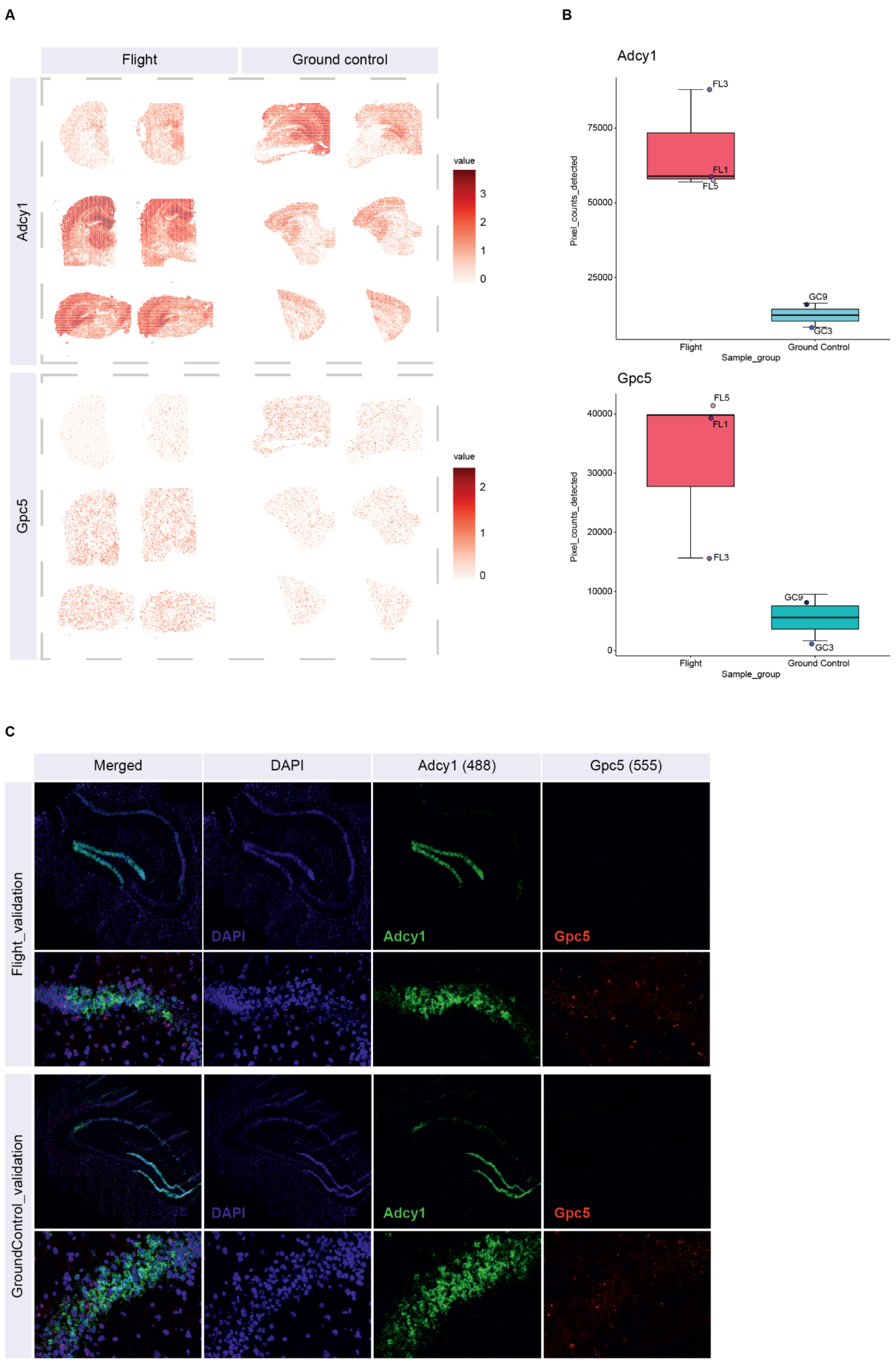

Supplementary Figure 13: A, Spatial distribution of genes Adcy1 and Gpc5 across flight mouse brain samples (F1-F3) and ground control mouse brain samples (G1-G3) in the ST dataset. **B,**

Boxplots showing quantification of the signal for Adcy1 and Gpc5 genes detected across whole hemispheres of three flight samples and two ground control samples using the RNAscope method. The quantification was done by counting the pixels detected for each gene. FL1, FL3, FL5 - Flight; GC3, GC9 - Ground control. **C,** RNAscope fluorescence image of one flight and one ground control mouse brain samples in hippocampal region visualizing nuclei (blue, DAPI) and genes Adcy1 (green, 488) and Gcp5 (red, 555). Each row immediately below these images, for each group, represents the close-up view of the same hippocampal region at 40X resolution. Adcy1 (green, 488) was found to be upregulated in the hippocampal CA3 (ST cluster 8) and dentate gyrus regions (ST cluster 11) of the spaceflight samples.
