# Supplementary material for: Spatially resolved multiomics on the neuronal effects induced by spaceflight in mice
Source: Nat Commun. 2024 Jun 11;15:4778. doi: 10.1038/s41467-024-48916-8 (PMC11166911; doi:10.1038/s41467-024-48916-8)
Supplement: Supplementary file 3 — Description of Additional Supplementary Information [file 41467_2024_48916_MOESM3_ESM.pdf]

Description of Additional Supplementary Data

Filename: Supplementary Data 1

Description: Multiomics cluster marker genes. Marker genes identified for each of the 18 clusters identified in the multiomics clusters of RR3 brain data using two-sided Wilcoxon rank sum test.

Filename: Supplementary Data 2

Description: Multiomics clusters annotations. Functional annotations of the 18 (0-17) multiomics clusters identified in the multiomics brain dataset according to functions. Cluster Annotation column denotes the shorter names for these cluster annotations. Celltype annotation column refers to the celltype assignments for each multiomics cluster.

Filename: Supplementary Data 3

Description: Differentially expressed genes (DEGs) for multiomics clusters. DEGs identified for the 18 multiomics clusters in the spaceflight samples of RR3 brain data using two-sided Wilcoxon rank sum test.

Filename: Supplementary Data 4

Description: Overlapping spaceflight DEGs. A set of 11 overlapping spaceflight DEGs between the single nuclei multiomics and bulkRNAseq data of the same mice from the NASA RR-3 mission. Genes (Gabra6, and Kctd16) with the same directional change are in italicized text.

Filename: Supplementary Data 5

Description: Overlapping known spaceflight DEGs. This table lists all the datasets that were included in the comparison of known spaceflight DEGs (comparison between Flight vs Ground Control) with the 825 multiomics spaceflight DEGs found in the multiomics RR-3 brain data. A hypergeometric distribution test was performed to test the overlap significance.

Filename: Supplementary Data 6

Description: ST clusters annotation. Categorization of the 18 (0-17) ST clusters identified in the spatial dataset according to their spatial location and function.

Filename: Supplementary Data 7

Description: ST cluster marker genes. Marker genes identified for the 18 spatial (ST) clusters in RR3 brain data using two-sided Wilcoxon rank sum test.

Filename: Supplementary Data 8

Description: DEGs for ST clusters. Differentially expressed genes for spaceflight samples found across the 18 ST RR3 brain clusters using two-sided Wilcoxon rank sum test.

Filename: Supplementary Data 9

Description: Deconvolution results using Stereoscope. ST clusters that matched with celltype proportions (multiomics clusters) found by deconvolution using Stereoscope.

Filename: Supplementary Data 10

Description: The list of 1260 LR pairs found across the spatial data using SpatialDM. Each column value corresponds to the z-scores that were later used in the differential testing.

Filename: Supplementary Data 11

Description: List of 134 differential ligand-receptor pairs found in the spatial dataset. Differential testing was done between flight and ground control groups using likelihood ratio test and threshold of differential p-value < 0.1.

Filename: Supplementary Data 12

Description: Motif analysis. Top 10 motifs analysed for multiomics clusters using ATACseq peaks. Motifs repeated in multiple clusters are highlighted in italics font. Two-sided wilcoxon rank sum test was done to estimate for p-values.

Filename: Supplementary Data 13

Description: Metabolic analysis results for ST dataset. FGSEA results with adjusted p-values for spatial (ST) RR3 brain clusters. A two-sided Wilcoxon Rank Sum test was done to estimate for p-values.

Filename: Supplementary Data 14

Description: Metabolic analysis results for multiomics dataset. FGSEA results with adjusted p-values for multiomics (snRNA-seq and snATAC-seq combined) RR3 brain clusters. A two-sided wilcoxon rank sum test was done to estimate for p-values.
