# Supplementary material for: Spatially resolved multiomics on the neuronal effects induced by spaceflight in mice
Source: Nat Commun. 2024 Jun 11;15:4778. doi: 10.1038/s41467-024-48916-8 (PMC11166911; doi:10.1038/s41467-024-48916-8)
Supplement: Supplementary file 18 — Reporting Summary [file 41467_2024_48916_MOESM18_ESM.pdf]

nature portfolio

Corresponding author(s): Stefania Giacomello

Last updated by author(s): Apr 15, 2024

Reporting Summary

Nature Portfolio wishes to improve the reproducibility of the work that we publish. This form provides structure for consistency and transparency in reporting. For further information on Nature Portfolio policies, see our Editorial Policies and the Editorial Policy Checklist.

Statistics

For all statistical analyses, confirm that the following items are present in the figure legend, table legend, main text, or Methods section.

n/a	Confirmed	
☐	☒	The exact sample size (n) for each experimental group/condition, given as a discrete number and unit of measurement
☐	☒	A statement on whether measurements were taken from distinct samples or whether the same sample was measured repeatedly
☐	☒	The statistical test(s) used AND whether they are one- or two-sided<br>*Only common tests should be described solely by name; describe more complex techniques in the Methods section.*
☐	☒	A description of all covariates tested
☐	☒	A description of any assumptions or corrections, such as tests of normality and adjustment for multiple comparisons
☒	☐	A full description of the statistical parameters including central tendency (e.g. means) or other basic estimates (e.g. regression coefficient) AND variation (e.g. standard deviation) or associated estimates of uncertainty (e.g. confidence intervals)
☐	☒	For null hypothesis testing, the test statistic (e.g. F, t, r) with confidence intervals, effect sizes, degrees of freedom and P value noted<br>*Give P values as exact values whenever suitable.*
☒	☐	For Bayesian analysis, information on the choice of priors and Markov chain Monte Carlo settings
☐	☒	For hierarchical and complex designs, identification of the appropriate level for tests and full reporting of outcomes
☐	☒	Estimates of effect sizes (e.g. Cohen's d, Pearson's r), indicating how they were calculated

Our web collection on statistics for biologists contains articles on many of the points above.

Software and code

Policy information about availability of computer code

Data collection

RNA was extracted from tissue sections using the RNeasy Mini kit (Qiagen). RNA integrity was measured by using the Agilent Bioanalyzer.

Spatial gene expression libraries were generated using the Visium Gene Expression kit (10x Genomics) and prepared following the manufacturer's protocol (User Guide, CG000239 Rev F). The resulting libraries were sequenced by using Illumina Novaseq and Illumina NextSeq500 platforms.

Nuclei were extracted by following 10XGenomic protocol for Single Cell Multiome ATAC + Gene Expression Sequencing (User Guide, CG000375 Rev B). Cell sorting was done utilizing a BD Bioscience Influx flow cytometer using an 86 µm nozzle following gating strategy: nuclei:singlets:7-AAD positive events. BSA coated Eppendorf tube was used to collect the nuclei. The resulting libraries were sequenced by using Illumina NextSeq2000 platform.

Data analysis

ST data (count matrices) were processed using Space Ranger software (version 1.0.0, 10X Genomics) with alignment done with the built-in mouse reference genome (mm19-3.0.0). ST data analysis was done in R (v4.1.1) using the packages: Seurat (v4.0.1) and STUtility (v0.1.0) in R.

Multiomics count matrices were generated using CellRanger ARC (v2) software from 10X Genomics with mapping done from mouse genome, mm10 reference 2020-A from 10X Genomics. Data analysis was done in R (v4.1.1) using packages Seurat (v4.1.0) and Signac (v1.6.0).

Additionally, JASPAR (v2020_0.99.10) was used along with ChromVAR (v1.16.0) for motif analysis. lme4 (v1.1-2.9000) package was also used in motif analysis for chromatin accessibility scores. EnrichR and Consensus Pathway Analysis paltform were used for pathway analysis.

CellPhoneDB (v3) and SpatialDM (v0.1.0) were used for ligand-receptor analysis. CellOracle, PROGENy and MISTy models were leveraged for the spatial pattern analysis to identify correlations between pathway activities and for differential interactions identification.

fgsea (v1.22.0) package in R (v4.2.1) was used for metabolic pathway analysis.

Shinyapp was designed in R (v4.1.1) using package shiny (v1.7.1) using theme superhero. R package babelgene (v22.9; R version 4.3.2) was used to identify human orthologues for genes displayed in the shinyapp.

Validation experiments were performed using Single molecule Fluorescence In Situ Hybridization (smFISH) RNAscope technology following manufacturer's protocol (Advanced Cell Diagnostics, ACD, Hayward, CA). Visualization of target genes was done using HRP-based RNAscope Fluorescent Multiplex Assay V2 (Cat. No. 323110). Quantification of the fluorescence signal was done with Fiji in ImageJ software and the images were taken with a ZEN LSM700 confocal microscope exported as tiff with the same settings.

All data used during and generated from the analysis are deposited to NASA OSD-352 (https://doi.org/10.26030/jm59-zy54) study page, Mendeley Data repository (https://10.17632/fjxrcbh672.1), and Figshare project (https://doi.org/10.6084/m9.figshare.24581544).

All scripts to reproduce the analysis as well as generate the figures presented in this study are provided in our publicly accessible Github repository (https://github.com/giacomellolab/NASA_RR3_Brain).

For manuscripts utilizing custom algorithms or software that are central to the research but not yet described in published literature, software must be made available to editors and reviewers. We strongly encourage code deposition in a community repository (e.g. GitHub). See the Nature Portfolio guidelines for submitting code & software for further information.

Data

Policy information about availability of data

All manuscripts must include a data availability statement. This statement should provide the following information, where applicable:

- Accession codes, unique identifiers, or web links for publicly available datasets
- A description of any restrictions on data availability
- For clinical datasets or third party data, please ensure that the statement adheres to our policy

The raw data generated in this study is hosted on the NASA GeneLab server, a part of the NASA Open Science Data Repository under study ID OSD-352 (https://doi.org/10.26030/jm59-zy54). This includes raw data (fastq files and brightfield images) from the ST and multiomics datasets, as well as processed data (aggregated gene count matrices, metadata, and fragment files) from the multiomics dataset. GeneLab, as an integral component of NASA's open-science initiative, provides researchers with raw and processed multi-omics data from spaceflight and ground-based analogue experiments, fostering broad scientific collaboration. Additionally, processed count matrices generated for the ST samples, the final data generated from the analysis performed in this study, and the source data for the generated figures are publicly accessible from Mendeley dataset (https://doi.org/10.17632/fjxrcbh672.1). Furthermore, the RNAscope validation images with genes Adcy1 and Gpc5 for all the validated tissue sections are available on Figshare (https://doi.org/10.6084/m9.figshare.24581544).

Research involving human participants, their data, or biological material

Policy information about studies with human participants or human data. See also policy information about sex, gender (identity/presentation), and sexual orientation and race, ethnicity and racism.

Reporting on sex and gender	n/a
Reporting on race, ethnicity, or other socially relevant groupings	n/a
Population characteristics	n/a
Recruitment	n/a
Ethics oversight	n/a

Note that full information on the approval of the study protocol must also be provided in the manuscript.

Field-specific reporting

Please select the one below that is the best fit for your research. If you are not sure, read the appropriate sections before making your selection.

☒ Life sciences ☐ Behavioural & social sciences ☐ Ecological, evolutionary & environmental sciences

For a reference copy of the document with all sections, see nature.com/documents/nr-reporting-summary-flat.pdf

Life sciences study design

All studies must disclose on these points even when the disclosure is negative.

Sample size	No sample size calculation was done.<br>The availability of spaceflown samples from NASA was limited, hence the sample size was subjected to availability.<br>However, consecutive sections was taken from each mice sample in the Visium experiments to increase sample size and to reflect the reproducibility of the data capture. The whole hemisphere information was used in the nuclei isolation experiments to maximize data yield.<br>Specific data analysis methods were employed in order to extract robust biological findings, for example, MAST with mixed-model approach was used in the differential expression analysis.
Data exclusions	No data was excluded
Replication	Multiple sections from each mouse brain sample were taken for reproducibility. For Visium, consecutive sections from each brain sample were run under the same experimental conditions. In the case of nuclei isolation experiments, one brain sample was used to optimize the protocol and then all remaining samples were run under the same conditions following the optimized protocol. The multiomics samples were generated using the Single Cell Multiome ATAC + Gene Expression Sequencing (User Guide, CG000375 Rev B) following the standard manufacturer's protocol. For both Visium, and mutiomics experiments, gene expression correlation tests were performed to test for gene expression capture agreement between the samples within the conditions as well as across conditions. Good correlation scores were observed between different mouse samples confirming the reproducibility of the experiments.
Randomization	No randomization was performed.<br>Mice of similar age, sex and strain were used as ground controls housed in identical hardware (Rodent Habitat AEM-X, identifier 1379) and matching ISS environmental conditions, including but not limited to cage type, light cycle (standard 12:12 light/dark cycle for both ground control and flight mice groups), food (Nutrient Upgraded Rodent Food Bar (NuRFB)), temperature (23.36 ?, and 21.37 ? on average for ground control and flight mice, respectively), humidity (41.86% and 41.53% on average for ground control and flight mice, respectively) and CO2 concentration (3664.89 ppm mean CO2 for ground controls, and 3711.97 ppm mean CO2 for flight mice).
Blinding	No blinding was done. The results do not include subjective measurements and were performed by individual researchers.

Reporting for specific materials, systems and methods

We require information from authors about some types of materials, experimental systems and methods used in many studies. Here, indicate whether each material, system or method listed is relevant to your study. If you are not sure if a list item applies to your research, read the appropriate section before selecting a response.

Materials & experimental systems

n/a	Involved in the study	
☒	☐	Antibodies
☒	☐	Eukaryotic cell lines
☒	☐	Palaeontology and archaeology
☐	☒	Animals and other organisms
☒	☐	Clinical data
☒	☐	Dual use research of concern
☒	☐	Plants

Methods

n/a	Involved in the study	
☒	☐	ChIP-seq
☒	☐	Flow cytometry
☒	☐	MRI-based neuroimaging

Animals and other research organisms

Policy information about studies involving animals; ARRIVE guidelines recommended for reporting animal research, and Sex and Gender in Research

Laboratory animals	Mus musculus. Strain: BALB/c (Age: 12 weeks; Sex: Female) and B6129SF2/J (Age: 14-15 weeks; Sex: Female).<br>Mice of similar age, sex and strain were used as ground controls housed in identical hardware (Rodent Habitat AEM-X, identifier 1379) and matching ISS environmental conditions, including but not limited to cage type, light cycle (standard 12:12 light/dark cycle for both ground control and flight mice groups), food (Nutrient Upgraded Rodent Food Bar (NuRFB)), temperature (23.36 ?, and 21.37 ? on average for ground control and flight mice, respectively), humidity (41.86% and 41.53% on average for ground control and flight mice, respectively) and CO2 concentration (3664.89 ppm mean CO2 for ground controls, and 3711.97 ppm mean CO2 for flight mice).
Wild animals	No wild animals were used in this study.
Reporting on sex	All Female BALB/c mice (for both groups: spaceflight and ground controls) and B6129SF2/J mice (for both groups: spaceflight and ground controls).
Field-collected samples	No field-collected samples.

Ethics oversight	The study followed recommendations in the Guide for the Care and Use of Laboratory Animals and the protocol (CAS-15-001-Y1) was approved by the NASA Flight Institutional Animal Care and Use Committee (IACUC) for both flight (housed at the NASA Ames Research Center) and ground control (housed at the Kennedy Space Center) mice.

Note that full information on the approval of the study protocol must also be provided in the manuscript.

Plants

Seed stocks	n/a
Novel plant genotypes	n/a
Authentication	n/a
